# Opioids in Treatment of Refractory Dyspnea in Chronic Obstructive Pulmonary Disease: Yes, No or Maybe

**DOI:** 10.3390/jpm14030318

**Published:** 2024-03-19

**Authors:** Ruxandra-Mioara Rajnoveanu, Antonia Harangus, Doina Adina Todea, Milena Adina Man, Corina Eugenia Budin, Armand-Gabriel Rajnoveanu

**Affiliations:** 1 Palliative Medicine Department, Iuliu Hatieganu University of Medicine and Pharmacy, 400012 Cluj-Napoca, Romania; ruxandra.rajnoveanu@umfcluj.ro; 2 Leon Daniello Pulmonology Hospital, 400012 Cluj-Napoca, Romania; 3 Research Center for Functional Genomics, Biomedicine and Translational Medicine, Iuliu Hatieganu University of Medicine and Pharmacy, 400012 Cluj-Napoca, Romania; 4 Department of Medical Sciences—Pulmonology, Iuliu Hatieganu University of Medicine and Pharmacy, 400012 Cluj-Napoca, Romania; dtodea@umfcluj.ro (D.A.T.); adina.man@elearn.umfcluj.ro (M.A.M.); 5 Department of Pathophysiology, George Emil Palade University of Medicine, Pharmacy, Science, and Technology of Târgu Mureș, 450142 Targu Mures, Romania; corina.budin@umfst.ro; 6 Occupational Medicine Department, Iuliu Hatieganu University of Medicine and Pharmacy, 400012 Cluj-Napoca, Romania; armand.rajnoveanu@umfcluj.ro

**Keywords:** opioid, treatment, refractory dyspnea, chronic obstructive pulmonary disease

## Abstract

Chronic Obstructive Pulmonary Disease (COPD) is a complex condition with significant impact on prognosis, especially in advanced stages where symptom burden becomes critical. Breathlessness affects patients’ quality of life, and despite various therapeutic strategies, the role of opioids in palliative care for COPD remains under investigation. The acceptance of a therapeutic trial of different types of opioids is increasing not only in end-of-life situations but also for stable COPD patients experiencing intolerable refractory breathlessness despite optimal conventional therapy. Recent clinical trials have raised questions about the overall clinical benefit of opioids in addressing breathlessness in COPD, prompting the need to clarify inconsistencies and identify specific subgroups that may benefit from opioid therapy. In the clinical setting, it is crucial to understand the attributes of patients who exhibit positive responses to opioids and what type of opioids could have a positive impact. This research paper aims to offer an update of the most recent evidence of opioid treatment in managing breathlessness among individuals with COPD with a head-to-head evaluation of the supporting and opposing proof in the medical literature.

## 1. Background

Chronic Obstructive Pulmonary Disease (COPD) is a diverse pulmonary condition caused by chronic airway inflammation and/or alveolar destruction, leading to persistent, often progressive, airflow obstruction [1]. The intricate underlying pathophysiology exerts a substantial impact on both prognosis and health status, where health status is defined as the influence of health on the capacity to engage in and get satisfaction from daily activities, encompassing health-related quality of life and functional status [2]. The management of COPD presents a complex challenge, particularly in the advanced stages where symptom burden and quality of life become critical considerations. Among the various therapeutic options, the role of opioids in palliative care for COPD has been a subject of ongoing debate and investigation [3]. As the prevalence of COPD continues to rise globally, there is a need for effective interventions to alleviate symptoms, especially dyspnea. 

The term “chronic breathlessness syndrome” or “refractory dyspnea” has been recently defined, referring to a condition characterized by breathlessness experienced either at rest or during physical exertion, resulting in functional impairment despite receiving optimal medical care. Another pattern of breathlessness is the acute, episodic “breakthrough” breathlessness [4]. In individuals with advanced chronic respiratory diseases such as COPD, breathlessness stands out as the primary and incapacitating symptom [5,6]. The persistence of breathlessness has substantial consequences for the quality of life of patients and contributes to considerable psychological distress [7]. Breathlessness serves as a significant predictor of exercise tolerance. Quantification of breathlessness can be accomplished directly using scales like the modified Borg Scale, Visual Analogue Scale (VAS), and Numeric Rating Scale (NRS) or indirectly by assessing its influence on physical activity, as demonstrated by the modified Medical Research Council (mMRC) breathlessness scale [8]. Alleviating dyspnea during daily activities to minimize disability, enhance quality of life, and reduce the utilization of medical resources is a primary objective in the care of individuals with COPD. Various therapeutic strategies can be explored to address the diverse mechanisms at play. These encompass the predominant use of inhaled bronchodilators, self-management education, where patients acquire breathing techniques, and participation in pulmonary rehabilitation programs that incorporate exercise training [1].

There is a limited options of pharmacological treatments in chronic breathlessness syndrome with well-established evidence for overall clinical benefit. However, analyses combining data from predominantly smaller trials have indicated a positive impact on breathlessness using systemic (non-nebulized) opioids, such as morphine [9,10]. There are high concentrations of opioid receptors in the brain stem, where they primarily exert an inhibitory effect on respiratory drive, largely mediated by μ-opioid receptors. Additionally, opioids possess anxiolytic properties that contribute to the alleviation of dyspnea and may mitigate the impact of arterial carbon dioxide and oxygen levels on ventilation, ultimately decreasing oxygen consumption during periods of rest [11]. 

Recent randomized clinical trials have cast doubt on the overall clinical advantage of opioids in addressing chronic breathlessness [12,13,14]. The variability in these findings may pose a challenge to recommending opioids for alleviating breathlessness, particularly in non-cancerous conditions. To substantiate the role of opioids in treating breathlessness, a clarification of the reasons behind these inconsistencies is essential. Additionally, it is crucial to define the specific subgroups that respond positively to opioid therapy. 

This study aims to review the latest available evidence on the use of opioid treatment for individuals experiencing chronic breathlessness in the context of COPD. Navigating the palliative landscape for COPD also demands a thorough examination of patient and healthcare provider perspectives. This research paper seeks to add insights to the ongoing discourse on the role of opioids in COPD by informing clinical practice and guiding future research endeavors.

## 2. Materials and Methods

Studies were identified through extensive research of three electronic databases (Pubmed, MEDLINE, and EMBASE). Limitations were set at January 2000 and 8 January 2024 to obtain not only the latest evidence but also to identify the main studies published during the last twenty years from reviews, randomized controlled trials, clinical trials, systematic reviews, or meta-analysis. The key words used in various combinations with different Boolean operators were “opioids”, “COPD”, “chronic obstructive pulmonary diseases”, “refractory dyspnea”. There were no restrictions on text availability, article type, or language. Following the database analysis, a manual search of other web articles and journals was performed. 

## 3. Rationale for the Use of Opioids in COPD

The historical evolution of opioid use in COPD reflects a changing paradigm in the approach to symptom management. Early reservations about the potential risks associated with opioids have gradually given way to a nuanced understanding of their benefits, particularly in the palliative care setting [15]. The physiological rationale for the use of opioids in COPD lies in the modulation of the respiratory drive, offering a potential avenue for relieving dyspnea. The analgesic effects and respiratory depression induced by opioids result from the activation of μ-opioid receptors (MORs). These receptors are expressed in neurons responsible for regulating breathing, primarily situated in the brainstem. Specifically, MORs are prominently found in the Nucleus Tractus Solitarius (NTS), Retrotrapezoid Nucleus (RTN), and Median Raphe Nuclei (MRN) [16]. In principle, opioids could alleviate dyspnea by potentially reducing minute ventilation and/or reducing the respiratory response to chemoreceptor stimulation induced by hypoxemia and hypercapnia [17,18,19,20]. 

MORs are additionally situated in the deeper regions of the cerebral cortex, such as the thalamus, or anterior cingulate cortex. Opioid activity at these sites might play a role in modulating the perception of dyspnea [19,20]. Moreover, opioids present anxiolytic properties that extend beyond their respiratory effects, contributing to an overall reduction in the perception of breathlessness [21]. As anxiety and depression intensify, the response to opioids seems to diminish. Therefore, patients with notable depression and/or anxiety should initially undergo non-pharmacological psychoeducational interventions to alter the influence of emotions on opioid responsiveness and mitigate the potential adverse effects resulting from unwarranted dose escalation [22].

The current understanding about the perception of breathlessness stems from neuroimaging studies, primarily from utilizing functional magnetic resonance imaging (fMRI) [22]. The brain seems to establish expectations and beliefs, subsequently shaping the perception of incoming sensations of breathlessness. Consequently, individuals experiencing chronic breathlessness process this sensation within the context of memories and fears associated with past experiences. Opioids seem to have an effect on reducing the anticipatory dyspnea [22,23]. The individual response to morphine might be related to the existence of the single nucleotide polymorphism on the HTR3B gene [24], but prospective studies are needed to validate this. Understanding the intricate interplay of these mechanisms forms the foundation for exploring the effectiveness of opioids in mitigating the symptom burden of COPD.

## 4. Type of Opioids: Extended-Release Formulations and Immediate Delivery Agents

The literature outlines two approaches to prescribing opioids for refractory dyspnea: short-acting and long-acting agents [25,26]. The later were first used officially for this purpose in Australia, where extended-release oral morphine (Kapanol) has expanded its license to include, for the first time globally, the indication for the pharmacological treatment of chronic breathlessness [22]. The clinical trials investigating opioids for breathlessness in COPD patients have focused on both short-acting agents [27,28] and extended-release formulations [9,12], but despite this, there have been no direct comparisons or head-to-head assessments of the two opioid prescribing approaches. There is considerable uncertainty regarding the appropriate timing and methodology for prescribing either of the methods to alleviate breathlessness. For individuals with impaired swallowing or those in the advanced stages of end-of-life care, transmucosal, transdermal, subcutaneous, nebulized, or intravenous administration may be more suitable [29]. 

Nebulized opioids delivered directly to the airways showed modest and limited benefit on breathlessness over time [9]. Only opioids with lipophilic properties can be absorbed through intranasal or intraoral mucosa [29]. The rationale for this alternative route is the presence of opioid receptors within the epithelium of trachea and large bronchi on unmyelinated C nerve fibers and pulmonary neuroendocrine cells (PNEC) [30]. Directly targeting these receptors has a potential clinical benefit for dyspnea and for lowering some important side effects like constipation and dizziness when compared to the systemic morphine, and this motivates the ongoing research on the nebulized use of opioids in COPD [31]. The use of 3–5 mg morphine delivered by a dosimetric nebulization system was effective in reducing chronic breathlessness intensity in 10 severe COPD patients, while maintaining a good safety profile [31]. The role of a particular type of nebulization system such as the dosimetrically operated nebulizer, seems to be highly important, as only 10% of the set dose reaches the lungs when using jet nebulizers [32]. 

Despite the overall scarcity of evidence on this topic, the nebulized use of opioids other than morphine was tested. The study of Jensen et al. [33] on 12 stable mild-to-severe COPD patients using a single-dose inhalation (50 mcg) of fentanyl citrate showed significant and potentially clinically important improvements in exercise tolerance. Still, no extrapolation can be made as the sample size was small and further studies to evaluate the benefit of inhaled fentanyl in COPD patients are lacking [33]. Fentanyl has also been studied in the form of nasal spray for the relief of breathlessness in patients with COPD [34,35], as it has a bioavailability of approximately 70% and its onset of action occurs rapidly, typically between 12 to 22 min after administration [36].

Regarding the short-acting morphine, these molecules could play a significant role in alleviating exertional breathlessness [37]. In a study involving opioid-naïve patients, immediate-release oral morphine sulfate at a dose of 0.1 mg·kg^−1^ of body mass, with a maximum dosage of 10 mg has demonstrated beneficial effects [27]. An alternative approach includes the administration of immediate-release liquid morphine with an initial dosage of 0.5 mg twice daily. This may be followed by potential weekly adjustments based on clinical reassessment, and there is the option of eventually transitioning to a sustained-release formulation [38,39]. 

Utilization of sustained-release morphine for refractory dyspnea has been argued to represent the superior prescribing strategy among the two. This is based on the claim that shorter-acting opioid formulations tend to have less consistent steady-state blood levels. This, in turn, heightens the potential for suboptimal responses and adverse effects [40,41]. The approach usually involves the administration of a sustained-release oral morphine tablet once daily, ranging from 10 to 30 mg [12,14,42].

## 5. Evidence Supporting the Use of Opioids in COPD

As mentioned earlier, there is a theoretical rationale [16,17,18,19,20] supporting the use of opioids for managing refractory dyspnea in COPD, and there is existing evidence, although limited, that supports the effectiveness of opioids for this purpose. Until 2020, only three meta-analyses of randomized, double-blind, placebo-controlled trials investigating opioid therapy for refractory dyspnea have been conducted [9,29,43].

Systemic opioids, commonly employed for refractory pain, were proposed to alleviate refractory breathlessness, as indicated by a meta-analysis of 18 small trials by Jennings et al. This is one of the first meta-analyses published on this subject, and it included a subgroup of 14 trials involving COPD patients. The cumulative count of patients enrolled in the oral or parenteral studies amounted to 116, whereas the total number of patients encompassed in the nebulized studies was 177. The meta-analysis indicated a statistically significant positive impact of systemic opioids on the perception of breathlessness (*p* = 0.0008), and the COPD subgroup had comparable results to the main analysis [43]. However, no clear impact on exercise capacity was identified. Meta-regression revealed a more pronounced effect in studies employing oral or parenteral opioids compared to those utilizing nebulized opioids (*p* = 0.02). The difference in the impact of nebulized opioids compared to nebulized saline may have been affected by the fact that the latter can also influence breathlessness through the stimulation of facial nerve endings and the liquefaction of secretions [43]. 

More recent data also indicated a slight to moderate reduction in breathlessness with systemic opioids in patients with COPD [9]. The meta-analysis encompassed 16 studies, comprising 15 crossover trials and 1 parallel-group study, with a total number of 271 participants, 95% of whom had severe COPD (n = 258 patients). In the main analysis, systemic opioids demonstrated a positive effect on breathlessness in outpatients measured at a steady state (five studies, ninety-one participants): Standardized Mean Difference (SMD) 20.33 (20.52 to 20.14; I^2^, 30.4%). The study found no concurrent enhancement in exercise performance and, due to the heterogeneity of the studies and inadequate data, a meta-analysis of health-related quality of life (HRQL) and adverse effects could not be conducted [9]. The main limitations were the brief duration of the studies and the limited information on the prolonged effects of opioids. Additionally, there was a scarcity of data regarding the variation in effects based on the doses administered (dose-response) and the distinctions between various types of opioids. The GRADE-rated evidence for nebulized opioids was considered low, emphasizing the need for additional pharmacological and clinical studies to validate the net clinical benefit for breathlessness.

A more extensive systematic review was conducted by Barnes et al. [29], involving 26 studies and 526 participants (each study with less than 50 subjects), of which fourteen studies predominantly or exclusively included participants with COPD. There was a large variety in opioid types and dosages: dihydrocodeine doses varied between 15 mg three times a day and 60 mg three times a day, diamorphine was administered at doses ranging from 2.5 to 5 mg four times a day, while sustained-release morphine was used in each of the included studies in daily doses of 10 to 20 mg. Nebulized morphine doses showed a wide range, from 1 mg to 50 mg daily. The authors tried to determine the morphine dose equivalent that would provide relief from breathlessness. However, the presence of substantial heterogeneity among trials precluded the identification of a clear dose threshold. The study concluded that there was low-quality evidence indicating the benefit of oral or parenteral opioids. The study did not reveal any statistically significant difference in breathlessness when comparing nebulized or intravenous morphine with a placebo. Although the change from baseline (in six studies) did not show significant differences, post-treatment scores (from 12 studies) favored opioids over placebo (standardized mean difference (SMD) −0.32, 95% confidence interval (CI) −0.53 to −0.10). Morphine and dihydrocodeine exhibited a substantial treatment effect [29]. 

The idea that opioids can alleviate breathlessness and increase exercise capacity in severe COPD patients received further support from a crossover trial conducted by Abdallah et al. in a laboratory setting [27]. This trial involved 20 individuals with severe (stage 3 or 4) COPD, with mMRC ≥ 3, and where the administration of a maximum dose of 10 mg of oral immediate-release morphine (compared to a placebo) resulted in a decrease in both exertional ventilation and breathlessness (by a mean of 1.2 ± 0.4 Borg units). These measurements were taken at a standardized time point (iso-time) during cardiopulmonary exercise testing. Ratings for the intensity and unpleasantness of breathlessness were comparable between treatments at the end of exercise. The enhancements in exertional breathlessness and exercise endurance induced by morphine were accompanied by slight, yet statistically significant, reductions in ventilation and breathing frequency during exercise at iso-time. The researchers suggested that opiates might alleviate dyspnea by modifying central sensory signaling and reducing activity in corticolimbic centers through opioid receptor blockade. Importantly, there were no significant opioid-related side effects. These findings may have limited applicability due to a small and homogenous cohort of clinically stable individuals with severe-to-very severe COPD [27].

Professional societies advocate for the use of opioids in the context of breathlessness in COPD [1,36,39,44,45]. In this regard, the study conducted by Rocker et al. [38] offered a deeper understanding of the experiences of patients dealing with advanced COPD and severe refractory dyspnea (mMRC ≥ 4) when using immediate-release morphine sulfate syrup. The long-term impact of the therapy on health-related quality of life, dyspnea, anxiety, and depression were also assessed. Although it had a small sample size (44 patients), the study concluded that opioid treatment proved beneficial for 61% of patients with advanced COPD experiencing dyspnea who did not respond to conventional therapy. Positive effects were observed early on and, for most patients, were maintained over an extended period of several months [38].

## 6. Latest Supporting Updates—Subgroup Analysis

The MORDYC study [12], published in 2020, is a randomized, double-blind, placebo-controlled intervention involving 111 patients with COPD and moderate to very severe breathlessness. The participants were administered 10 mg of standard oral sustained-release morphine or a placebo twice daily for a duration of 4 weeks, with the option to escalate the dosage to three times daily after 1 or 2 weeks. The results demonstrated that oral extended-release (ER) morphine was clinically and statistically significant in improving disease-specific health status, as measured by the COPD Assessment Test (CAT). The morphine group exhibited a 1.19 mmHg higher difference in PaCO_2_ (95% CI, −2.70 to 5.07 mmHg; *p* = 0.55), and there was no notable change in breathlessness. Notably, participants with mMRC grades 3 to 4 experienced an improvement in worst breathlessness, showing a 1.33-point decrease in the morphine group (95% CI, −2.50 to −0.16 points; *p* = 0.03). There was a significant difference in respiratory rate between the treatment groups, with an advantage observed in favor of morphine [12]. Another sub-analysis on patients with an mMRC score exceeding 3, from a trial that included 58% (n = 164) patients with COPD and chronic breathlessness, also confirmed that the numerical rating scale (NRS) or visual analog scale (VAS) for the worst breathlessness significantly decreased in those administered opioids compared to the placebo group [42]. These newly emerged data could also influence the practice of prescribing opioids for advanced, oxygen-dependent COPD. In a Swedish study that included 2249 patients with COPD, only 2% of the patients were prescribed opioids to alleviate breathlessness, while 97% received opioids for pain [46].

In a further attempt to find specific COPD patients that might benefit from opioids, a recent cross-sectional analysis published in 2022 [47] examined the association between a clinically significant improvement (defined as ≥1 point on a 0–10 numeric rating scale) in the moderate-to-very severe chronic breathlessness of 45 COPD patients and baseline variables, including sensory breathlessness descriptors, age, breathlessness, and body mass index (BMI). The study found that a greater baseline breathlessness and a higher body mass index (BMI) are linked to a clinically meaningful improvement in breathlessness among patients using 20–30 mg of oral sustained-release morphine for four weeks. The subgroup benefit was also proven in one of the most recent randomized controlled trials (RCT) involving regular, low-dose extended-release morphine in COPD, known as the BEAMS (Breathlessness, Exertion, And Morphine Sulfate) trial that included 156 participants [14]. Even if the trial did not demonstrate improvement in the primary outcome (worst breathlessness after 1 week of treatment), it suggested the existence of ‘super-responders’ within the participant group, those patients who reported the intervention to be life-changing, with noted improvements such as easier breathing, better sleep, increased engagement in meaningful activities, and overall higher well-being. The improvements in dyspnea scores, active minutes, and active calories utilized persisted after 6 months of morphine use. The research projected that a sample size of 135 individuals would yield 80% statistical power to identify a minimal clinically important difference. [14].

The most recent meta-analysis, published by Liu et al. in 2023 [48], confirmed some of the findings presented above. The paper included 24 studies, involving 975 patients. Crossover studies revealed that opioids significantly improved breathlessness (standardized mean difference, −0.43; 95% CI, −0.55 to −0.30; I^2^ = 18%) and exercise endurance (standardized mean difference, 0.22; 95% CI, 0.02–0.41; I^2^ = 70%). However, in parallel control studies administering sustained-release opioids for more than 1 week, opioids did not show improvement in dyspnea and exercise endurance. The authors concluded that sustained-release opioids did not demonstrate improvement in dyspnea and exercise endurance, while short-acting opioids, though appearing safe, showed the potential to be used as a prophylactic treatment for exertional dyspnea [48].

**Figure 1:**
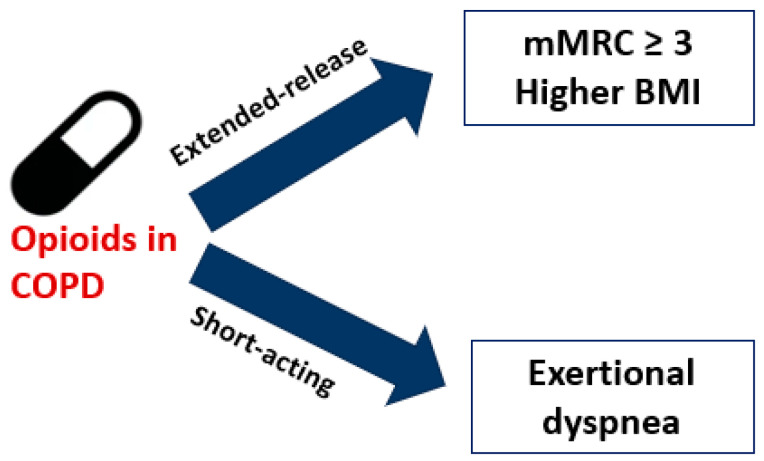
Specific subgroups of opioid responders to immediate or extended-release opioid formulations.

As proved by the studies presented above, the use of opioids proved beneficial as an intervention for refractory dyspnea in certain individuals with advanced COPD. A noteworthy number of patients sustained these benefits for months, aligning with recent suggestions to contemplate the use of opioids in certain subgroup of patients with different phenotypes (higher BMI; mMRC ≥ 3) or outcomes (exertional dyspnea), as presented in Figure 1.

Continued research is exploring the effectiveness of opioids, aside from morphine, in alleviating dyspnea among COPD patients. Persistent concerns regarding potential side effects and respiratory insufficiency serve as obstacles in the prescription of morphine by pulmonologists. The lack of a placebo controlled RCT investigating the benefits of transdermal fentanyl on breathlessness in COPD patients was a strong rationale for Dijk and her team to start, in 2019, the MoreFoRCOPD study (NCT03834363) [49]. It is a multi-center, double blind, double-dummy, crossover, randomized, placebo-controlled clinical trial on sixty patients with severe stable COPD and refractory dyspnea (FEV_1_ < 50%, mMRC ≥ 3, on optimal standard therapy) that compares morphine (sustained-release 10 mg twice a day) or transdermal fentanyl (12 mcg/h) for refractory dyspnea in COPD or placebo. The study will be completed in 2024, so no data are available now [49].

## 7. Evidence against the Use of Opioids in COPD

In a particular set of trials with the primary objective of reducing breathlessness, opioids showed no effectiveness in patients with COPD [12,13,14,42]. Table 1 summarizes the findings of the main studies presented in the pro and con chapters regarding the benefit of opioids in COPD.

The randomized controlled trial (RCT) by Currow et al. [42], consisting of 284 individuals with a predominant presence of COPD (58%) and experiencing moderate to severe breathlessness (mMRC ≥ 2), revealed no discernible impact of 20 mg oral extended-release (ER) morphine over a period of seven days in both the primary outcome of alleviating breathlessness and health-related quality of life. Similarly, another treatment group comprising 155 participants demonstrated a comparable lack of advantage when administering 15 mg oral ER oxycodone over the course of 1 week as opposed to a placebo [13]. A limitation of these investigations was the accessibility of ‘as needed’ morphine to all participants, with slightly higher usage observed in the placebo groups [13,40]. Furthermore, a systematic review that included fourteen studies found no significant change in dyspnea at rest or post-exercise, with no significant improvement in exercise capacity [50]. 

The majority of the above-mentioned studies [13,14,42] ensured 80% power to detect a clinically meaningful difference between the groups in the primary endpoint. In the context where treatment with opioids did not yield significant improvements in COPD patients with severe chronic breathlessness, potential sources of type II errors (false negatives) might have played a role. First, some of the studies had a relatively small sample size and it may have lacked the statistical power necessary to detect a true effect. The chosen dosing regimen and duration of treatment could also be factors contributing to type II errors. Moreover, the heterogeneity of the COPD population, unexplored subgroups that might respond differently to opioids, and the relatively short duration of the studies could further contribute to overlooking potential benefits. Careful consideration of these factors is essential when interpreting the outcomes. 

In 2013, Simon et al. [51] conducted a systematic review on fentanyl for relieving refractory breathlessness that also included patients with COPD (18 in total), identifying two before–after studies, nine case studies, and two RCTs (one with only two participants). While descriptive studies consistently reported breathlessness relief with fentanyl, a pilot RCT (n = 12) [33] did not reveal statistically significant improvements compared to placebo. The studies covered various fentanyl administration methods, including intranasal, nebulized, oral transmucosal, transdermal, and intravenous. Therefore, until now, the overall scientific evidence supporting the use of fentanyl nasal spray for breathlessness treatment remains limited.

The use of opioids has been associated with a range of psychomotor and gastrointestinal side effects, which may include dizziness, delirium, somnolence, falls and fractures, constipation, nausea, and vomiting. These effects are particularly noteworthy among older adults [41,52,53,54]. Observational studies on various populations have reported an association between opioid use and increased adjusted mortality [55]. However, it is noteworthy that the use of symptom-control medications, including opioids, tends to rise with age and proximity to death [56]. This suggests that opioids may serve as a marker of advancing illness rather than being a direct cause of death [55,56].

Opioids may pose respiratory risks for individuals with COPD through various potential mechanisms: depression of the central respiratory drive [17,57]; activation of opioid receptors in the tracheobronchial tree, leading to cough suppression and subsequent mucous impaction [58]; potential aspiration due to sedation; and negative immunomodulatory effects [59,60]. These potential adverse respiratory effects of opioids are particularly pertinent for individuals with COPD, given their compromised baseline respiratory function and susceptibility to recurrent acute respiratory exacerbations. Observational data regarding the connections between opioid treatment and adverse events in patients with COPD have shown inconsistent and conflicting results. In 1981, Woodcock et al. conducted the inaugural clinical study on opioids for alleviating breathlessness in severe COPD and first emphasized the importance of cautious monitoring and patient selection due to the associated risks of respiratory depression and addiction [61]. In the subsequent four decades, various randomized, double-blind studies have investigated the efficacy of opioids in managing breathlessness among individuals with COPD [9,12,27]. These studies, overall, indicate that low-dose opioids yield a modest yet clinically significant enhancement in chronic breathlessness without elevating the risk of respiratory depression or carbon dioxide retention. However, it is important to note that many of these studies had a very brief duration.

Ekstrom et al. [62] discovered a link between higher opioid doses and heightened mortality in oxygen-dependent COPD patients. Prescriptions for opioids (1417 in total) encompassed weak opioids, such as tramadol (31% of all opioids), codeine (19%), and dextropropoxyphene (15%), as well as strong opioids, including oxycodone (15%), morphine (11%), and fentanyl (5%). The dose-response relationship between opioids and mortality revealed that lower doses (≤30 mg oral morphine equivalents per day) showed no association with increased mortality (1.03, 0.84 to 1.26), unlike higher doses of opioids (1.21, 1.02 to 1.44) [62]. Another retrospective analysis by Vozoris et al. included 89,327 COPD patients who received oral or transdermal opioids [63]. The authors reported that users of longer-acting opioid-only agents at a dose of ≤30 mg morphine equivalents per day exhibited a significantly increased risk of hospitalizations for COPD or pneumonia, as well as elevated mortality rates related to COPD or pneumonia and all causes; among those using longer-acting opioid-only agents at a dose of >30 mg morphine equivalents per day, there was a reduced risk of outpatient exacerbations but an increased risk for hospitalizations and mortality associated with COPD or pneumonia, as well as all-cause mortality [63]. On the other hand, retrospective data regarding opioid dependence and abuse in COPD patients revealed that these can elevate the risk of intubation in hospitalized COPD patients without impacting mortality. No variations were observed in this study in the use of non-invasive ventilation (NIV) or length of stay [53]. 

Nevertheless, retrospective population-based studies face inherent limitations, such as the inability to ascertain the specific reason for opioid use and to match controls for the severity of the treated individuals’ disease. Given that refractory dyspnea commonly prompts opioid use, and that dyspnea is closely correlated with mortality in COPD, the absence of adjustment for dyspnea severity in the analyses conducted by Ekstrom et al. [62] and Vozoris et al. [63] poses a significant risk of confounding by indication [64,65,66]. In both studies, it remains possible that opioid use merely indicated the presence of refractory dyspnea and signaled the severity of the underlying disease.

Apart from the adverse effects related to the pharmacological class, opioids can have several drug interactions with other COPD medications. Like beta-agonists, inhaled muscarinic antagonists do not undergo metabolism through the cytochrome P450 system and they do not exhibit significant pharmacokinetic interactions. The primary adverse effects of inhaled muscarinic antagonists, particularly at elevated doses, involve local and systemic anticholinergic effects [67]. When administered via inhalation, muscarinic antagonists are often used in conjunction with tricyclic antidepressants, antipsychotics, antisecretory medications, and certain antiemetics. This combination may lead to clinically significant adverse effects such as dry mouth, constipation, or blurred vision. Opioids, which may also cause constipation and urinary retention, can have these effects exacerbated by excessive anticholinergic activity resulting from high dose inhaled muscarinic antagonists. Additionally, the combination of muscarinic antagonists may heighten tachycardia, a side effect associated with nabilone [67].

Considering the existing evidence, careful evaluation and consideration of individual patient characteristics, symptomatology, and potential interactions are crucial when contemplating the use of opioids in COPD management.

## 8. Current Guidelines and Future Endeavors

Prudent utilization of opioids in the context of advanced COPD is endorsed by various respiratory guidelines [1,39,44,46,68]. The American Thoracic Society [44] suggests considering opioid-based therapy for managing advanced refractory dyspnea in individuals with COPD within a personalized shared decision-making approach. The Canadian Thoracic Society [39] recommends using oral opioids for treating refractory dyspnea in advanced COPD patients, while the American College of Chest Physicians [68] states that the principle of double effect justifies using opioids or sedatives to relieve dyspnea, even if it may hasten death in patients with advanced lung disease.

However, these guidelines emphasize the necessity for data regarding the overall clinical benefits and safety of opioids in this population [1,39,44,46,68]. Due to the limited sample sizes in most of the studies presented above, conducting larger trials with over 50 participants per treatment arm could enhance the robustness of evidence for the use of opioids in managing breathlessness. Consideration should be given to randomized, parallel-group trials of extended durations for increased clinical relevance. Moreover, efforts should be directed towards identifying effective dosing schedules to achieve the maximum therapeutic effect with minimal side effects, while incorporating standardized outcome measures, including consistent assessments of breathlessness and quality of life, to ensure uniformity in study outcomes.

Despite the endorsement of systemic low-dose opioids for alleviating refractory breathlessness in advanced COPD by clinical practice guidelines in Canada, the United States, Europe, and internationally, a considerable number of physicians refrain from prescribing opioids for breathlessness. This reluctance is often attributed to concerns about potential adverse side effects, a perceived lack of robust scientific evidence supporting the efficacy of opioids in treating refractory breathlessness, and the challenge of predicting which patients will positively respond to opioid therapy [27]. The stigma associated with opioid use results in their underutilization, causing many patients with advanced COPD to endure prolonged and severe dyspnea unnecessarily as they approach the end of life. 

## 9. Conclusions

While consistent evidence supporting efficacy of opioids in alleviating breathlessness in COPD is lacking, the absence of a definitive demonstration of ineffectiveness and reports of potential benefits in certain individuals exist. In the diverse range of studies exploring opioid use in COPD and among various opioid formulations (short-acting or long-acting) and administration routes (oral, nebulized, intranasal, intravenous), the most substantial evidence supports the use of sustained-release morphine for managing refractory dyspnea in patients experiencing dyspnea rated at mMRC ≥ 3 and individuals with a higher BMI. The immediate effects of morphine on exertional dyspnea were observed in studies involving ergometer and treadmill activities. The suggested, safe dosage is currently ≤30 mg per day orally. 

There is insufficient evidence to suggest that any specific opioid substance, form, or route of administration has a superior impact on breathlessness compared to others. Favorable outcomes were observed in descriptive studies investigating the effectiveness of fentanyl in alleviating breathlessness, but efficacy trials are lacking. Additional research is required to elucidate the optimal prescription approach to minimize adverse effects for both oral, nebulized, and intranasal routes of administration. Opioid titration should be done gradually to strike a balance between side effects and achieving an individualized target for refractory dyspnea.

## Figures and Tables

**Table 1:** Summary of the findings of the main studies presented in the pro and con chapters regarding the benefit of opioids in COPD (other than meta-analysis/systematic reviews).

Study/Analysis	Number of Participants /mMRC	Opioid Type	Dosage	Breathlessness Scale	Outcome Measure	Findings
Janoviak et al. [31]	10 (mMRC ≥ 3)	Nebulized morphine	3–5 mg	VAS	Chronic breathlessness now	Superiority of dosimetrically administered nebulized morphine.
Abdallah et al. [27]	20 (mMRC ≥ 3)	Oral immediate-release morphine	10 mg maximum dose	Borg	Exertional breathlessness	Morphine decreased breathlessness intensity ratings during exercise at isotime.
Rocker et al. [38]	44 (mMRC ≥ 4)	Immediate-release morphine sulfate syrup	0.5 mg twice daily (initially)	NRS and Chronic Respiratory Questionnaire–Dyspnea domain	Health-related quality of life, dyspnea, anxiety, depression	Beneficial for 61% of patients; positive effects early on and maintained over several months.
MORDYC study [12]	111 (mMRC ≥ 2)	Oral sustained-release morphine	10 mg twice daily (escalation to three times daily)	NRS	Disease-specific health status (COPD Assessment Test), PaCO_2_, Breathlessness	Improvement in disease-specific health status; no notable change in breathlessness; improvement in worst breathlessness for mMRC grades 3 to 4.
Currow et al. [42]	164 (mMRC ≥ 2)	Oral sustained-release morphine	20 mg daily (≤6 doses of 2.5 mg immediate-release morphine (≤15 mg/24 h)	VAS	Intensity of breathlessness now	No difference between groups. Decreased worst breathlessness in opioid subgroup (for mMRC ≥ 3) compared to placebo.
Verberkt et al. [47]	45 (mMRC ≥ 3)	Oral sustained-release morphine	20–30 mg	NRS	Chronic breathlessness	Greater baseline breathlessness and higher BMI linked to improvement.
BEAMS trial [14]	160 (mMRC ≥ 3)	Oral sustained-release morphine	Week 1: 8 mg/d or 16 mg/d or placebo Week 2–3: +8 mg/d	NRS	Intensity of worst breathlessness	No difference between groups.
MoreFoRCOPD study [49]	Ongoing (mMRC ≥ 3)	Oral sustained-release or transdermal fentanyl	10 mg twice a day (morphine) or 12 mcg/h transdermal fentanyl	NRS	Refractory breathlessness	Ongoing
Jensen et al. [33]	12	Nebulized fentanyl citrate	50 mcg	Borg	Exercise tolerance and dyspnea intensity and unpleasantness	Increased exercise endurance time. Delay in the onset of intolerable dyspnea.

mMRC = modified Medical Research Council; VAS = visual analog scale; NRS = numeric rating scale; BMI = body mass index.

## Data Availability

No new data were created or analyzed in this study. Data sharing is not applicable to this article.
